# Integrin Regulation in Immunological and Cancerous Cells and Exosomes

**DOI:** 10.3390/ijms22042193

**Published:** 2021-02-23

**Authors:** Zay Yar Soe, Eun Jeong Park, Motomu Shimaoka

**Affiliations:** 1 Department of Physiology, University of Medicine, Magway, 7th Mile, Natmauk Road, Magway City 04012, Magway Region, Myanmar; 2 Department of Molecular Pathobiology and Cell Adhesion Biology, Mie University Graduate School of Medicine, 2-174 Edobashi, Tsu-City 514-8507, Mie, Japan; epark@doc.medic.mie-u.ac.jp

**Keywords:** integrin, exosome, talin, kindlin, endothelial metabolism, angiogenesis

## Abstract

Integrins represent the biologically and medically significant family of cell adhesion molecules that govern a wide range of normal physiology. The activities of integrins in cells are dynamically controlled via activation-dependent conformational changes regulated by the balance of intracellular activators, such as talin and kindlin, and inactivators, such as Shank-associated RH domain interactor (SHARPIN) and integrin cytoplasmic domain-associated protein 1 (ICAP-1). The activities of integrins are alternatively controlled by homotypic lateral association with themselves to induce integrin clustering and/or by heterotypic lateral engagement with tetraspanin and syndecan in the same cells to modulate integrin adhesiveness. It has recently emerged that integrins are expressed not only in cells but also in exosomes, important entities of extracellular vesicles secreted from cells. Exosomal integrins have received considerable attention in recent years, and they are clearly involved in determining the tissue distribution of exosomes, forming premetastatic niches, supporting internalization of exosomes by target cells and mediating exosome-mediated transfer of the membrane proteins and associated kinases to target cells. A growing body of evidence shows that tumor and immune cell exosomes have the ability to alter endothelial characteristics (proliferation, migration) and gene expression, some of these effects being facilitated by vesicle-bound integrins. As endothelial metabolism is now thought to play a key role in tumor angiogenesis, we also discuss how tumor cells and their exosomes pleiotropically modulate endothelial functions in the tumor microenvironment.

## 1. Integrin Regulation in Cells

Integrins are cell adhesion receptors whose proper functioning plays a very significant role in diverse physiologic processes, such as embryonic development, cell proliferation, leukocyte adhesion and platelet aggregation [1]. As the name implies, integrins constitute the integral components of the plasma membrane, thereby integrating the extracellular and intracellular events of a living cell and transmitting the cellular signals bidirectionally [2]. In this review, we discuss the general activation mechanism of integrins in cells. We then describe the integrin-regulation mechanism in exosomes, as well as the biological functions and significance of exosomes derived from immunological and cancerous cells. As these exosomes modulate the behavior, metabolism and functions of endothelial cells, we elucidate their role in tumor angiogenesis.

### 1.1. Structures of Integrins

Structurally, integrins are type I transmembrane glycoproteins that consist of non-covalently associated α and β subunits. Eighteen α subunits and eight β subunits combine to give rise to 24 different α/β heterodimeric integrins [3]. Different combinations of integrin subunits confer specificity not only in binding to extracellular and cellular ligands but also in transmitting intracellular signals. For instance, the integrins αLβ2/leukocyte function-associated antigen (LFA)-1, αMβ2, α4β1 and α4β7 are principally expressed in leukocytes and the defined interactions of αLβ2 and αMβ2 with intercellular adhesion molecule 1 (ICAM-1), of α4β1 with vascular cell adhesion molecule 1 (VCAM-1) and of α4β7 with mucosal addressin cell adhesion molecule 1 (MAdCAM-1). Indeed, they are important for leukocyte adhesion to the endothelium and for T-cell homing [4,5]. 

Either the α or β subunit of integrins comprises an extracellular, globular domain (ectodomain), a single-pass transmembrane domain (TMD) and a short cytoplasmic tail (CT) (except for β4 integrin) [1]. The extracellular domain is responsible for interactions with integrin ligands that are present on cells (e.g., ICAM-1, VCAM-1 and MAdCAM-1), the extracellular matrix (ECM) (e.g., fibrinogen, collagen and laminin) and micro-organisms (e.g., β-glucan on *Candida albicans*) [5]. The structural dynamics and stability of TMD and CT are critical for integrin activation. This is exemplified by platelet integrin αIIbβ3, whose TMD dimers are packed in stable, right-handed, helix–helix interactions and stabilized by the formation of outer and inner membrane clasps. It is thought that αIIb TMD is embedded in a straight orientation, while β3 TMD adopts a 25°-tilted angle to the plane of the lipid bilayer. Non-covalent heterodimeric interactions between α or β CT (e.g., electrostatic interaction between Arg995 of αIIb and Asp723 of β3) consistently maintain integrin in its resting or inactive state. Consequently, the separation of TMD and CT gives rise to integrin activation [3,6,7].

**Figure 1:**
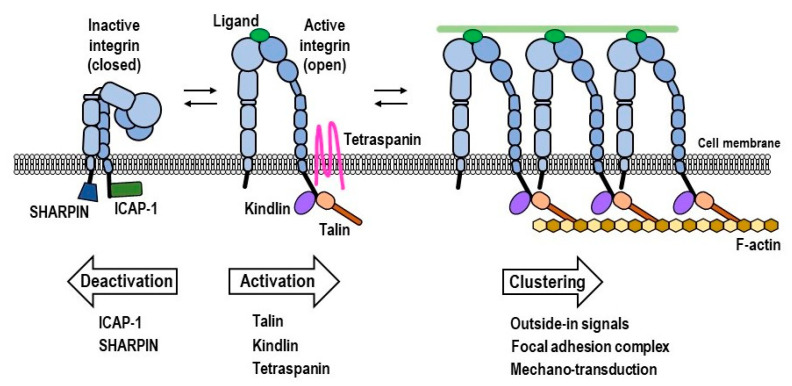
Conformational regulation of integrins on the cell surface. The inactive integrin adopts a closed conformation, in which the headpiece is folded back to the legpiece (right). Such an inactive integrin is stabilized by the association of the αβ cytoplasmic domain, which is strengthened by SHARPIN and ICAP-1. The binding of talin and kindlin induces dissociation of the αβ cytoplasmic domain, thereby triggering a global conformational change of the extracellular part to the active open integrin conformational state (middle). Tetraspanin associates with the integrin β subunit and modulates integrin activation. Extracellular ligation by cognate ligands and intracellular linkage to F-actin via talin facilitate the lateral association of integrin molecules, thereby enhancing cell adhesion.

The conformational transition of integrins from a default low-affinity state to an activated high-affinity state is tightly regulated (Figure 1). The ectodomain’s active conformational state is induced by an inside-out activating signaling cascade of cytoplasmic enzymes or proteins, which culminates in inducing the dissociation of constrained αβ CT. In immune cells, the inside-out signaling processes are initiated from cell-surface T-cell receptors or cytokine receptors. Upon high-affinity engagement of the activated integrin with its ligand counterpart, outside-in signals are transduced to the cytoplasm, leading to integrin clustering and modulation of intracellular pathways that are critical for altering cell spreading, migration, proliferation, differentiation and metabolism [1,2,3,7].

### 1.2. Intracellular Integrin Activators: Talin and Kindlin

To date, talin has been identified as the critical cytoskeletal protein (~270 kDa) involved in the final step of integrin activation, as binding of talin to the integrin CT induces the dissociation of αβ CT, thereby leading to the global conformational changes that result in active high-affinity integrin [8] (Figure 1). Talin is abundant in the cytoplasm, associates with integrins upon activation and mechanically links ECM components to cytoskeletal structures (Figure 1). Vertebrates express two isoforms of differently encoded talin molecules, talin-1 and talin-2, both exhibiting 74% homology of amino acid sequences [9]. The general structure of talin consists of a large, globular, N-terminal head domain (~50 kDa) and a long, flexible C-terminal rod domain (~220 kDa), linked by a calpain2-cleavage site. The talin head resembles FERM (C-terminal 4.1, erzin, radixin, moesin) domain structures and, unlike other FERM proteins, its F0, F1, F2 and F3 subunits are linearly arranged. The rod domain, which consists of 13 helical bundles (R1–R13), has binding sites for vinculin and actin [9] (Figure 1).

The current understanding of talin-mediated integrin activation began with studies examining platelet integrin αIIbβ3. These earlier studies reported that the overexpression of the talin head fragment could increase the ligand affinity for αIIbβ3 integrins and activate integrin in Chinese hamster ovary (CHO) cells [10]. Later, it was shown that the F3 subunit of the talin head, via its phospho-tyrosine (PTB)-like domain, binds with high affinity to the membrane proximal (NPxY) region of the β CT, an engagement that induces integrin activation by disrupting the salt bridges formed between α and β CT [11,12]. Although the F1 and F2 head subunits do not directly interact with integrin CT, they are nonetheless needed to maintain the high-affinity state of integrins [9]. In addition, the electrostatic interaction that occurs between the cell membrane and the talin head is essential for integrin activation [7]. Importantly, in hematopoietic cells, Ras-related protein 1 (Rap-1) and Rap-1-GTP-interacting adaptor molecule (RIAM) are involved in integrin activation by recruiting talin to the plasma membrane [3]. The Rap-1/ RIAM axis is indispensable for activating integrin β2, although it is not significant in platelet integrin αIIbβ3 activation or hemostasis function [13].

In the resting state, talin adopts an autoinhibitory or closed form in which a rod domain forms a complex with the talin head F2-F3 subunit, thereby masking the integrin-binding site on F3 subunit [14]. Nuclear magnetic resonance (NMR) spectroscopy and mutagenesis studies have revealed that the talin rod segment R9 binds to the talin head F2-F3 and prevents the PTB-like domain in F3 from interacting with β CT [9]. This auto-inhibition configuration, in which the integrin-binding site in the talin head is masked by the talin rod, must, therefore, be resolved in order to allow the integrin–talin interaction, thereby inducing cytoplasmic unclasping and integrin activation. The masking effect of the talin rod is counteracted by a talin activator, phosphatidylinositol 4,5-bisphosphate (PIP2), which interferes with the interaction between the talin rod and the talin PTB-like domain [15]. Another mechanism by which talin autoinhibition is resolved can be found in platelets. The switch region 2 (SR2) of the G protein α subunit can bind to the talin head F3 subunit. In this manner, it can interfere with the talin head (F3)/talin rod (R9) interaction, which is responsible for maintaining talin in an autoinhibitory state [16]. 

Like talin, another FERM domain-containing protein, kindlin (~75 kDa), binds to integrin CT and activates integrin (Figure 1). It has three family members, namely, kindlin-1 (kindlerin), kindlin-2 (mitogen-inducible gene 2/mig-2) and kindlin-3, each isoform exhibiting 50–60% sequence homology and tissue-specific expression patterns [17]. Kindlins are composed of canonical cloverleaf-shaped FERM domains, which can be subdivided into a ubiquitin-like F0 domain, an F1 domain containing a lipid-binding loop region (F1L), a pleckstrin homologous (PH)-inserted F2 domain and a PTB-like F3 domain. The N-terminal F0 domain supports PIP2-enriched cell-membrane binding, integrin activation and kindlin localization to focal adhesions [17]. The polylysine motif in F1L and the PH-inserted F2 domain are necessary for kindlin binding to lipid membranes. The F3 domain of all kindlins binds to a highly conserved distal NxxY motif in the CT of β1, β2 and β3 integrins to induce their high-affinity state [18]. 

Kindlin-3, which is predominantly expressed in all hemopoietic cells, regulates the functions of leukocytes and platelets. Kindlin-3 deficiency in humans can present as leukocyte adhesion deficiency type III (LAD III) and bleeding disorders, whereas in mice, it causes perinatal death resulting from severe hemorrhaging, anemia and infections [3]. Kindlin-3 regulates α4β1-dependent firm adhesion of effector T-cells, as well as LFA-1-mediated T-cell adhesion and crawling under physiological shear force. Interestingly, this regulatory function, which has been demonstrated in an experimental autoimmune encephalomyelitis (EAE) mouse model, is remarkably important when brain vascular endothelial cells express low levels of integrin ligands ICAM-1 and VCAM-1 [19]. The distinct role of kindlin-3 in LFA-1 activation is seen in neutrophils. Both kindlin-3 and talin-1 are critical for CXCL-1-induced LFA-1 activation. Both are involved in the neutrophil recruitment cascade in which kindlin-3 is only required for neutrophil arrest, whereas talin-1 is required for both rolling and arrest [20]. Intriguingly, one recent study revealed that PH domain-mediated kindlin-3 recruitment towards the plasma membrane of neutrophils in response to IL-8 occurs before the conformational activation of LFA-1 in order to arrest neutrophils from rolling [21]. Another study suggested that integrin-linked kinase (ILK) plays an important role in kindlin-3-dependent LFA-1 activation in neutrophils. The chemokine-induced upregulation of ILK kinase activity has been shown to be required for the activation of kindlin-3 via protein kinase C (PKC)-α-mediated phosphorylation [22]. Another essential and noteworthy role played by kindlin-3 can be found in tumor-surveilling non-classical monocytes. Kindlin-3 deficiency has been shown to perturb the patrolling of non-classical monocytes towards tumor cells and their scavenging function in tumor environments [23]. 

It is now recognized that talin alone is not sufficient, but rather that the co-operation of kindlin is needed for the full activation of integrin. This co-operative activity between kindlin and talin, by interacting with distinct regions in integrin CT, has been reported in previous studies. The overexpression of kindlin-1 or -2 can enhance the binding ability of an overexpressed talin head to αIIbβ3 CT in CHO cells [3]. In platelets, kindlin-3 supports talin head binding to integrins, as well as integrin αIIbβ3 activation by directly interacting with paxillin via its F0 domain [24]. All-atom microsecond-scale molecular dynamic simulations of integrin αIIbβ3 developed by Haydari et al. showed that kindlin-2 enhances stronger binding of talin-1 to the membrane proximal region of the CT and assists talin-1-mediated integrin activation [25].

### 1.3. Intracellular Integrin Inactivators: SHARPIN and ICAP-1

Integrin function is fine-tuned not only by activators, such as talin and kindlin, but also by several inactivators that directly interact with either an α or β CT [26] (Figure 1). Shank-associated RH domain interactor (SHARPIN), a component of the linear ubiquitin chain assembly complex, negatively regulates integrin activity by interacting with α CT [27]. It is an adaptor protein that participates in tumorigenesis, metastasis, inflammation and immune responses [28]. Pull-down experiments revealed that SHARPIN associates with a highly conserved WKxGFFKR sequence in the CT of α1, α2 and α5 integrins, but not to β1 CT [26,27]. Furthermore, SHARPIN suppresses the endogenous activity of β1 integrins in cancer cells and primary leukocytes [27]. However, SHARPIN cannot suppress talin head-mediated β3 activation in αIIbβ3-expressing CHO cells [29]. The study by Gao et al. reported contradictory findings, in which SHARPIN directly interacted with β1 CT to inhibit the activity of α5β1 integrin in CHO cells [29]. It seems apparent that SHARPIN selectively binds to α or β CT, depending on the cell type and situation. Other integrin inhibitors that interact with α CT include mammary-derived growth inhibitor (MDGI), calcium and integrin binding protein 1 (CIB1), nischarin and serine-threonine phosphatase 2A (PP2A) [26].

The important integrin inactivators that bind to β CT are integrin cytoplasmic domain-associated protein 1 (ICAP-1) and filamin. ICAP-1 inhibits integrin activation by interacting with the proximal NPxY motif in the C-terminal region of β CT. A yeast two-hybrid screening revealed that ICAP-1 specifically binds to β1A CT, but not to β2, β3 or β5 CT [30]. ICAP-1 has been shown to become involved in biological functions, such as integrin-dependent cell migration [30] and focal adhesion disruptions [31], Rho-A-dependent endothelial contractility and fibronectin remodeling [32]. Filamin, an actin-crosslinking protein, can bind to β1A, β3 and β7 integrins, thereby modulating talin-dependent integrin activation. Its immunoglobulin-like domain 21 competes with talin for the binding of an overlapping site on the cytoplasmic β7 tail [33]. The interaction of filamin with integrin is, in turn, regulated by a cytoskeletal adaptor, migfilin, which can remove filamin from integrin-binding sites, thereby promoting talin–integrin interaction and integrin activation [34].

### 1.4. Force-Driven Integrin Activation

As mechano-sensing receptors, integrins can detect biophysical cues, such as ECM stiffness, shear stress, stretch and fluid pressure, in the external milieu of the cells, information which is relayed to cytoskeletal structures inside the cells [35]. For instance, α5β1 responds to ECM stiffness by forming α5β1-fibronectin adhesive bonds, a tensional force that correlates with substrate stiffness [36]. Mechanical force can regulate integrin conformational states. The dynamic equilibrium between the bent inactive and extended active forms of integrin αLβ2 on living cells is dynamically switched by force via an engaged ligand ICAM-1 [37]. Actin-driven traction force has been shown to be significant in full activation of integrin αLβ2 at the immunological synapse [38] as well as in migrating T-cells [39]. The application of tensional forces favors integrins to form catch bonds with their ligands, an adhesion phenomena, in which force prolongs bond lifetime [40]. Integrin–ligand engagement induces the accumulation of numerous proteins at the integrin CT to form focal adhesions at cell–cell or cell–ECM junctions. The most prominent proteins involved are talin and vinculin, which directly link the integrin to actomyosin [41]. Notably, force transmission on talin is dependent on actin flow. Actin flow is fast, and tensional force is low in small nascent adhesions, whereas talin tension becomes flow-independent in large adhesions [42]. The piconewton forces applied via integrins are finally transmitted to cytoskeletal actin, culminating in alterations of cytoplasmic protein synthesis, signaling pathways and cellular functions, such as tissue morphogenesis, migration, proliferation and differentiation. Dysregulation in integrin-ECM bidirectional mechano-transmission can give rise to many pathologic conditions, including cancer [41].

The activation of integrins has feedback effects on cellular tensional homeostasis. β1 integrin activation with its ligand fibronectin or stimulation with manganese increases traction forces, whereas β3 integrin exerts a negative effect on cellular tension [43]. The role played by the trans-dominant inhibition of integrins in mechano-transduction process has been reported. By modulating its affinity to kindlin, integrin αVβ3 negatively regulates the contractile forces generated by α5β1 integrin in mouse embryonic fibroblasts [44].

### 1.5. Heterotypic Lateral Association of Integrins with Tetraspanins and Other Membrane Proteins

Tetraspanins and syndecans (SDCs) are the prominent membrane proteins that laterally associate with integrin receptors in the same cell, thereby modifying integrin functions (Figure 1). Tetraspanins, such as CD9, CD63, CD81, CD82, and CD151, are a family of four-transmembrane-domain proteins with a large extracellular loop containing a highly conserved CCG motif and a small extracellular loop [45]. Tetraspanins and integrins form complexes on the surface of different cell types [45,46]. In addition, they modulate the functions of integrin-mediated cell adhesion, cell migration and downstream signaling, thereby regulating cellular behaviors, such as those observed in metastasis [47,48]. The regulation of integrin activity by tetraspanin–integrin association seems to be situation- or association-specific. CD9 is important in potentiating α5β1 integrin into the high-affinity state in CHO cells, thereby regulating α5β1-dependent cell motility to fibronectin via a phosphatidylinositol-3 kinase (PI3K)-mediated pathway [47]. In contrast, CD9 is not involved in regulating the affinity state of αLβ2 integrin, but does affect integrin clustering in T-cells, B-cells and monocytes [49]. The importance of the CD151–integrin α3β1 association is evident in human lung adenocarcinoma cells, human glioma cells and monkey kidney cells. CD151 modulates the stabilization of integrin α3β1’s active state and its binding capacity to its high-affinity ligand laminin [48]. In Jurkat cells, CD82 co-localizes with LFA-1 integrin, and its overexpression promotes LFA-1/ICAM-1-dependent T-cell adhesion, although the affinity modulation by CD82 remains unknown [50]. In particular, the involvement of CD81 in LFA-1-mediated T cell–B cell interactions does not likely stem from a direct CD81–integrin association [51].

SDCs belong to a family of membrane-associated heparan sulfate proteoglycans (HSPG). They are type I transmembrane proteins comprising a highly conserved short CT, a single-pass TMD and a less conserved large ectodomain with covalently attached heparin sulfate-glycosaminoglycan chains [52]. The physical or functional association between SDCs (except for SDC-3) and various kinds of integrins is well described [53]. Indeed, their crosstalk has been shown to affect cell adhesion [54,55], migration [56], cellular signaling [57], integrin recycling [58], angiogenesis [59] and tumor progression [60]. Of the four family members, SDC-1, via its regulatory site on the extracellular domain, directly associates with integrin αVβ3 or αVβ5 in mammary carcinoma cells [56] and vascular endothelial cells [60]. By lateral interactions with these integrins, SDC-1 regulates their activation state during cell spreading [56], cell migration [56], angiogenesis [60] and tumorigenesis [60]. The mechanism by which SDC-1 regulates integrin activity may be partly explained by another study, which found that the engagement of SDC-1 with integrin αVβ3 or αVβ5 activates insulin-like growth factor receptor 1 (IGFR1), thereby leading to autophosphorylation of IGFR1 and stimulation of the inside-out integrin-signaling pathway [61]. Conversely, the association between SDC-1 and the cytoplasmic domain of α6β4 in cancer cells regulates cell spreading and migration in a human epidermal growth factor receptor-2 (HER2)-dependent manner [62]. In addition to its physical association, a functional link between SDC-2 and integrin has also been reported. SDC-2 reduces the expression level of LFA-1 and locks LFA-1 in a low affinity state in Jurkat cells through the PDZ-binding domain of its CT, inhibiting the adhesion of these cells to human endothelial cells [55]. The extracellular portion of SDC-2 shed from the cell surface can inhibit integrin β1 activation and migration in nearby endothelial cells. These effects are mediated by the shed SDC-2 extracellular core protein, which engages with the tyrosine phosphate receptor CD148 expressed on endothelial cells [59]. SDC-4 interacts with integrin αVβ1 and endothelial surface molecule Thy-1 (CD-90), forming a cooperative trimolecular complex that uniquely displays this “dynamic catch”. This interaction has been implicated in force-induced cell adhesion [63]. The cooperation between SDC-4 and integrin β1 in mechanical adaption to force has also recently been reported. Localized tension applied on SDC-4 activates the PI3K/kindlin-2/integrin β1/Rho A axis, which is required for adaptive cell stiffening and remodeling of the ECM [64]. To date, the linkage of the largest SDC member SDC-3 with integrin remains poorly understood [52].

### 1.6. Homotypic Lateral Association of Integrins, Leading to Integrin Clustering

Integrin clustering is referred to as the oligomer formation of integrin heterodimers on the plasma membrane [7] (Figure 1). Integrin clustering modulates integrin avidity, i.e., it increases affinity-independent cellular adhesiveness, particularly in the presence of multivalent integrin ligands [1]. The clustering of integrin heterodimers plays a significant role in outside-in signaling; integrin recycling; and the formation of adhesion structures, such as nascent adhesions, focal adhesions and fibrillar adhesions, whereby numerous intracellular proteins, including integrin, talin and actin are recruited to initiate downstream signaling [7]. Notably, two essential integrin activator molecules, talin and kindlin, support integrin clustering. Deviating from a long-standing belief that the F2-3 domain of the talin head is essential for integrin clustering, one recent publication proposed a new mechanism underlying integrin β3 activation and clustering. The F1 loop of the talin head, via its interaction with the inner membrane clasp of integrin, appears to be important for mediating talin-dependent integrin activation and clustering [65]. 

Lateral diffusion of integrins in the cellular membrane is required for the rearrangement of integrin units into clusters [66]. In resting leukocytes, leukocyte-specific β2 integrins are in a low-affinity state and their mobility is constrained by numerous cytoskeletal components, such as actin microfilaments or microtubules. The depolymerization of actin microfilaments by cytochalasin D, or the disturbance of microtubule dynamics with nocodazole or taxol, enhances integrin mobility on the cell membrane and integrin-mediated cell adhesion [67]. It is assumed that upon integrin activation, they are released from these cytoskeletal restraints, thus enhancing their mobility and clustering abilities. This idea is supported by the study of Cluzel et al. in which the activation of integrin αVβ3 in mouse melanoma cells by Mn^2+^ or via the mutation of β3 was correlated to integrin clustering. Furthermore, immobilized ligands, talin and PIP2 are needed to facilitate integrin clustering [68]. In contrast, another study group reported intriguing results. On the surface of monocytes, integrin LFA-1 is preorganized as either resting nanoclusters or ligand-independent proactive nanoclusters (25%) that can readily bind to ligands. Upon conjugation with T-cells, only proactive nanoclusters are rapidly recruited to cell–cell contact sites in order to form micrometer-sized macroclusters and initiate binding to ICAM-1 [69]. Moreover, under physiologic cation conditions, these preorganized LFA-1 nanoclusters are mobile on the cell membrane of monocytes, while primed nanoclusters display static or free-diffusion behavior [66]. Therefore, the relationship between the conformational state, lateral mobility and clustering remains unclear, and whether these factors exert additive, sequential or independent effects on integrin activation remains a matter of debate. Of note, integrin clustering is also regulated by glycocalyx. It not only mediates integrin–ligand co-operation but also enhances integrin activation and clustering by shortening the physical distance between the cell membrane and ECM [35]. 

Thus far, we have explained integrin functions in cells. In the following sections, we describe the emerging role of integrins in exosomes.

## 2. Integrin Regulation in Exosomes 

### 2.1. Definition, Biogenesis and Function of Exosomes

**Figure 2:**
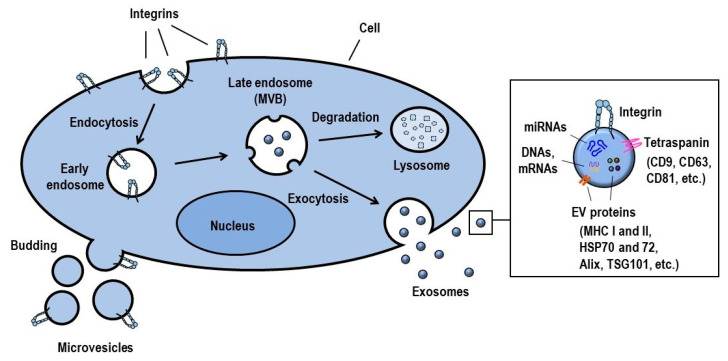
Biogenesis of extracellular vesicles. Exosomes are formed by the endosomal pathway; in this manner, they are secreted to the extracellular space. Microvesicles are released directly from the plasma membrane. Functional integrins are present on the surface of exosomes and microvesicles.

Exosomes are nanosized (40–120 nm), and comprise a lipid-bilayer-forming, heterogenous group of extracellular vesicles that are generated by almost all living cells, including immune cells and cancer cells (Figure 2). In addition, they can be detected in various biological fluids, such as blood, urine, saliva, cerebrospinal fluid, synovial fluid, breast milk, semen, intestinal secretions and bronchoalveolar lavage fluid [70]. Compared to cells, exosomes are approximately 100 times smaller, but their vesicular membranes display greater rigidity due to their higher proportion of sphingomyelin, desaturated lipids and cholesterol [71]. Although exosomes were initially regarded as cellular garbage vesicles, they are now believed to be important mediators in intercellular communication, as they can transport a variety of cargos, such as proteins, lipids and genetic materials (DNAs, mRNAs, miRNAs and non-coding RNAs) from donor cells to recipient cells (Figure 2). The contents loaded by exosomes vary depending on the physiologic conditions, cellular origins and biogenesis pathways. Specifically, inflammation, hypoxic conditions or acidic environments can modify the biochemical composition of exosomes being released [72]. The protein composition of exosomes includes cellular receptors (integrins and T-cell receptors), tetraspanins (CD9, CD63 and CD81), immuno-regulatory molecules (major histocompatibility complex/MHC I and II), cytoskeletal proteins (actin and tubulin), heat shock 70-kDa proteins (HSP70 and HSP72) and signal transduction molecules (G proteins and kinases). In addition, proteins related to exosome biogenesis (e.g., Alix and tumor susceptibility gene 101) are present in exosomes [73].

The biogenesis of exosomes starts with the inward budding of the endosomal membrane and the formation of intraluminal vesicles (ILV) in multivesicular bodies (MVB) (Figure 2). ILV formation is facilitated by many processes: high enrichment of CD9 and CD63 in the endosomal membrane, recruitment of the endosomal sorting complexes required for transport (ESCRTs) to the site of formation and interactions between syntenin and Alix. Sphingosine 1-phosphate is involved in the regulation of cargo sorting into exosomes. Upon fusion of the outer membrane of MVB to the plasma membrane, exosomes are released into the extracellular milieu. Rab GTPases, such as RAB11 and RAB35, facilitate this exosome release process. Lipid-modifying enzymes, such as neutral sphingomyelinase 2, phospholipase D3 and diacylglycerol kinase α, are also associated with exosome secretion. Once released, exosomes interact with recipient cell membranes and are endocytosed by target cells. In this way, exosome-loading cargos are liberated into the cytoplasm of target cells in order to induce cell signaling [72,74,75]. It is intriguing to note here that some integrin molecules initiate the formation of exosomes and determine the biological activities of exosomes. Integrin αMβ2 complex (complement receptor 3) is required for the formation and selective secretion of neutrophil-derived exosomes that exhibit bactericidal properties [76]. 

Exosomes have been implicated in both normal physiological phenomena and in pathological processes. In the heart, exosomes are important signals that can protect cardiomyocytes against injury or ischemia [77]. In the nervous system, they are crucial to neuro-glial communication and to the maintenance of neuronal activity under stressed conditions (e.g., starvation) [78]. In the developing embryo, exosomes serve as important mediators in maintaining the pluripotency of embryonic stem cells (ESCs) by engaging integrins via fibronectin and activating focal adhesion kinase (FAK) in ESCs [79]. Exosomes play a critical role in modulating immune responses. Dendritic cells prime naïve T-cells or downgrade their activity via exosomes, depending on their maturity [80]. The exosomal transfer of MHC protein complexes and antigens occurs between antigen-presenting cells [81]. T-cell exosomes influence the functions of T-cells [82], dendritic cells [83] and endothelial cells [84]. B-cell exosomes, which carry MHC II and co-stimulatory molecules, have the ability to present antigens to CD4^+^ T-cells [85]. NK cell exosomes, which carry cytolytic molecules and mediate cytotoxic cell lysis, are important in innate immunity [86]. Exosomes are involved in the pathogenesis of various diseases. For example, they have been linked to the progression of such neurodegenerative diseases as Alzheimer’s [87] and Parkinson’s diseases [88]. In particular, exosomes have been shown to participate in various steps of malignancy: tumor growth [89], tumor angiogenesis [90], inhibition of immunity [91] and metastasis [92]. The horizontal transfer of biological information, via exosomes, between cancer cells or between cancer cells and stromal cells/distant cells facilitates the creation of a pro-tumorigenic microenvironment [89,92,93,94]. The ability of tumor exosomes to alter endothelial cell characteristics, such as proliferation and migration, helps promote angiogenesis in the tumor environment. For example, soluble E-cadherin-positive exosomes derived from ovarian cancer cells interact with endothelial cells and enhance angiogenesis by activating β-catenin and the nuclear factor-κB (NFκB) signaling [90]. It seems likely that cancer exosomes favor the recruitment of tumor-suppressive cells and mediate immuno-suppressive effects probably by inhibiting the maturation of dendritic cells [91] and NK cell functionality [95] or by inducing the apoptosis of T-cells [96]. In addition, cancer-derived exosomes condition normal tissues to establish pre-metastatic niche. This function is supported by a study in which pancreatic ductal adenocarcinoma cell-derived exosomes induced pre-metastatic niche formation in the liver [97]. Their oncogenic function and ability to load different cargos that partly reflect the components of parent cells make exosomes potentially valuable biomarkers (exosomal proteins or miRNAs) for cancer diagnosis and prognosis [98,99]. Lastly, the ability of exosomes to confer drug resistance in tumor cells is important for cancer therapy [100].

### 2.2. Exosomal Integrin Functions

**Figure 3:**
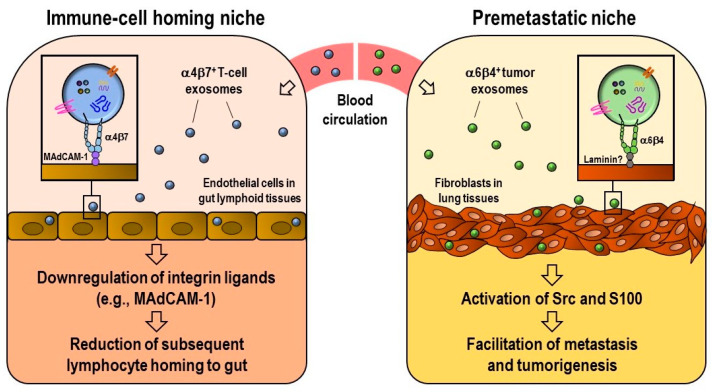
Exosomal integrin-mediated remodeling of the homing niches of immune cells (left) and cancer cells (right).

The inherence of integrins from cells to exosomes has an impact on the biological functions of cancer and immune cells (Figure 2). Exosomal integrins participate in multiple steps of cancer progression: they increase cell adhesion and migration, guiding cancer exosomes to specific metastatic sites (metastatic organotropism), preparing pre-metastatic niche formation, modulating the angiogenic potential of endothelial cells and activating the expression of certain genes (e.g., Src and the pro-inflammatory and pro-migratory S100 gene) [92,101,102]. Integrins exposed on exosomes engage with their specific ligands on target cells, and this interaction constitutes an important factor in the internalization of exosomes. Integrin α4β7-expressing T-cell exosomes make their entry into endothelial cells by engaging with ligand MAdCAM-1, which is expressed on the cells [84,103] (Figure 3). Exosomes are also key players in cell–cell communication by efficiently delivering integrins and associated kinases between similar or different cells in order to exert regulatory effects on recipient cells. For example, the integrins α2, αVβ3 and αVβ6 can be propagated between different prostate cancer (PrCa) cells. After accepting integrins, recipient cells acquire more aggressive cancer phenotypes [89,92,93,94,104]. Obviously, exosomes can serve as chemoattractant signals for the extravasation of neutrophils into inflamed tissues. Subramanian et al. found that the exosomes shed from neutrophils adhere to the endothelium in an integrin β2-dependent manner and locally release leukotriene B4 (LTB4), which is critical for neutrophil recruitment and extravasation in response to inflammatory signals [105]. The potential role of activated neutrophil exosomes in pulmonary diseases, such as chronic obstructive pulmonary disease and bronchopulmonary dysplasia, is also noted. ECM homeostasis in lung tissues is deranged by exosome elastase, which confers resistance to α1 anti-trypsin, and exosome integrin αMβ2, which mediates the lysis of ECM proteins [106]. In addition, the possible role of exosomal integrins in the pathogenesis of sepsis and systemic inflammatory response syndrome was reported by Kawamoto et al. Their study showed that exosomal integrin β2 expression in the plasma of septic patients is significantly higher than in that of healthy subjects and correlates with hypotension and reduced renal function [107]. The implication of exosomal integrins in conferring drug resistance and compromising drug efficacy has also been reported. One recent study showed that integrin α4β7-bearing circulating exosomes isolated from ulcerative colitis patients may mediate reductions in the therapeutic efficacy of the humanized monoclonal antibody vedolizumab, which is used for the treatment of inflammatory bowel disease. This occurs via the sequestration of vedolizumab, thus interfering with its binding to CD4^+^ T-cells [108].

### 2.3. Regulation of Integrin Function on Exosomes

Although the control mechanisms underlying integrin functions in cells have been studied for decades, the understanding of exactly how exosomal integrins are regulated remains in its infancy. To the best of our knowledge, we have demonstrated for the first time that, like other cellular systems, integrin function is regulated by talin in exosomes. TK-1 cells (i.e., a mouse T-lymphoma cell line) were used as a model to study the role of talin-2 in exosomes. We found that talin-2 deletion did not affect the vesicular expression of integrin α4β7 and LFA-1. However, talin-2 deletion significantly reduced not only the binding of exosomes to the integrin ligands MAdCAM-1 and ICAM-1 but also the uptake of exosomes by endothelial cells. Notably, the presence of talin-1 did not offset the talin-2 mediated effects observed in T-cell exosomes. These findings suggest that talin-2 regulates integrin-dependent functions in exosomes via a mechanism similar to that operating in cells [103]. Our findings warrant further investigation, in particular the functions of other integrin-regulatory molecules in exosomes. Integrin-binding partner proteins, such as kindlins and SHARPIN, are also expressed in cancer and immune cell exosomes (unpublished data). Thus, they may regulate integrin-mediated biological functions in exosomes.

In Section 1.5, we discussed in detail the lateral association of integrins with tetraspanins. It is obvious that tetraspanin proteins not only regulate cellular integrin activity and clustering but modulate integrin-dependent cell adhesion and migration. Tetraspanins (e.g., CD9, CD63 and CD81) are enriched components in the exosome membrane [109]. Increasing evidence has suggested that there is a co-expression of integrins and tetraspanins in the exosome compartment. For instance, tetraspanin 8 (Tspan8) selectively associates with integrin α4β1 in pancreatic cancer exosomes. Tspan8-α4β1 complex is required for exosomal interaction with endothelial cells and internalization to enhance tumor angiogenesis [110]. These results raise the possibility that exosomal integrin function would be controlled by tetraspanins, despite the need for further scientific evidence.

### 2.4. Integrin-Mediated Determination of the Biodistribution of Exosomes

The in vivo biodistribution pattern of exosomes is influenced by numerous factors: donor cell origin, route of administration, dose of injected exosomes, ligand recognition and targeted organs [111] (Figure 3). In a study conducted by Hoshino, they demonstrated that, when injected retro-orbitally into nude mice, exosomes isolated from a human breast cancer cell line (MDA-MB-231) and two pancreatic adenocarcinoma cell lines (BxPC-3 and HPAF-II) exhibited preferential distribution to the lung and liver, respectively. MDA-MB-231-derived exosomes were taken up three-fold more efficiently by the lung compared to BxPC-3 and HPAF-II exosomes. In contrast, the liver uptake of BxPC-3 and HPAF-II exosomes was four- to five-fold higher than that of MDA-MB-231 exosomes. This exosomal organotropism is thought to be mediated by the interaction of exosomal integrins and cell-associated ECM. Exosome proteomics, exosome uptake assays and integrin knockdown studies have confirmed that integrin α6β4 and α6β1 expression in breast cancer exosomes is associated with metastasis to laminin-rich lung microenvironments. In addition, integrin αvβ5 expression in pancreatic cancer exosomes has been linked to metastasis in fibronectin-rich liver microenvironments [101]. By this means, exosome-bound integrins play a critical role in guiding the distribution of tumor-derived exosomes to their specific destinations.

Similarly, our group showed the biodistribution patterns of immune cell exosomes. We specifically studied the role of exosomal integrin α4β7 in regulating the tissue distribution of T-cell exosomes. We found that integrin α4β7-expressing T-cell exosomes were well distributed in the small intestine, Peyer’s patches, the liver, spleen and mesenteric lymph node. However, β7-knockdown exosomes were not well distributed to the mucosa of the small intestine compared to control exosomes. This finding suggests that α4β7 is essential to promote the homing of T-cell exosomes to the small intestine [84].

### 2.5. Integrin-Mediated Internalization of Exosomes

Exosomes use multiple mechanisms (e.g., fusion, clathrin-mediated endocytosis, caveolae-mediated endocytosis, lipid raft-mediated endocytosis, macropinocytosis and phagocytosis) to gain entry into recipient cells [112]. Numerous studies have shown the importance of exosomal integrins to the communication that occurs between recipient cells and exosomes. Integrins present on exosomes help them dock on the surface of recipient cells. The exosome-bound integrins αVβ3 and α5β1 are involved in mediating the binding of exosomes to hepatic stellate cells [113]. B-cell exosomes express the integrins β1 and β2 on their outer surface, and these integrins help exosomes adhere to fibroblasts in order to elicit intracellular signaling events, such as Ca^2+^ signaling [114]. Although these studies did not carry out exosome uptake assays, exosomal binding to the cell surface is the initial and critical step for the subsequent internalization of exosomes into recipient cells.

Integrin-mediated exosomal uptake is believed to be important for communication between cancer cells and cancer metastasis. Integrin β3 (via interaction with HSPG) is crucial to the endocytosis of exosomes into breast cancer cells and exosome-induced colony formation [115]. The interaction between exosomal integrins and cellular ligands governs the selective uptake of cancer exosomes. Integrin α6β4-expressing breast cancer exosomes are preferentially taken up by lung fibroblasts, whereas integrin αVβ5-expressing pancreatic cancer exosomes are taken up by liver Kupffer cells. Subsequently, these exosomes upregulate the expression of proinflammatory S100 gene in their recipient cells for the preparation of premetastatic niche formation [101]. 

Exosomal integrins are also important to the uptake of exosomes by endothelial cells. T-cell exosomes use their surface-bound LFA-1 and α4β7 to enter mouse brain endothelial bEnd.3 cells, which constitutively express the integrin ligands ICAM-1 and MAdCAM-1 [103]. In addition, T-cell exosomes release their cargos, such as miRNAs, into recipient cells to suppress gene expression and to modulate the function of recipient cells [84]. Similarly, macrophage-derived exosomes can enter ICAM-1-expressing human brain endothelial cells via LFA-1. Neuroinflammation increases the uptake of these exosomes 3.1-fold by inducing the upregulation of ICAM-1 expression on brain endothelial cells. Furthermore, this study also demonstrated in vivo that naïve macrophage exosomes can cross the blood–brain barrier and ferry a cargo protein, brain-derived neurotrophic factor (BDNF), to the brain parenchyma, especially under inflammatory conditions [116]. It was shown that the binding of rat adenocarcinoma cell-derived exosomes to endothelial cells and subsequent internalization depend on the vesicular expression of the Tspan8-integrin α4 complex [110]. Collectively, these aforementioned studies demonstrated that integrin–ligand interactions are an important factor determining the cellular uptake of exosomes. Exosomal uptake is a prerequisite for the delivery of proteins and nucleic acids into recipient cells to alter cellular activities. A better understanding of exosomal integrin interaction with its target cells may provide an excellent opportunity for the delivery of targeted molecules to treat various diseases.

### 2.6. Exosomal Transfer of Integrin Proteins and Associated Kinases

The exosome-mediated transfer of bioactive molecules is a means to modulate the biological functions of recipient cells. The fate of recipient cells can be altered by exosomal integrins and associated kinases. The mechanism of selective packaging of integrins into exosomes by parent cells is not clearly understood. One study suggested that some proteins (e.g., integrin α4), which are about to be secreted in the exosome compartment, would be conditionally incorporated into tetraspanin-enriched detergent-resistant microdomains at MVB. This study indicated that tetraspanins are likely to be involved in the delivery of integrins into exosomes [117]. The exosomal integrin transfer in prostate cancer has been well demonstrated in numerous studies. Androgen receptor-positive PrCa cells exhibit aggressive features, such as increasing their proliferation, migration and invasion upon transfer of exosomal integrin α2 from castration-resistant cells [92]. Integrin αVβ3, which is implicated in aggressive cancers, is expressed in exosomes released by metastatic PrCa cells (PC3 and CWR22Pc) and is horizontally transferred to non-tumorigenic BPH-1 cells or other tumorigenic C4-2B cells. Both recipient cells then acquire an αVβ3-specific migratory phenotype. [89]. Another study group also reported that αVβ3 integrin is enriched in exosomes isolated from the blood of PrCa patients and that integrin αVβ3, via exosomes, can be transferred to αVβ3-negative PrCa cells in order to evoke a migratory phenotype [93]. Likewise, integrin αVβ6, which is not detectable in the normal human prostate, is highly expressed in PC-3 PrCa cells and can be transferred via exosomes to αVβ6-negative DU-145 PrCa cells. Upon exosome uptake, the recipient DU-145 cells display increased adhesion and migration on αVβ6-specific latency-associated peptides (LAP)-transforming growth factor β (TGFβ) [104]. Moreover, αVβ6-expressing PC3 exosomes can be transferred to β6-negative monocytes to induce M2 polarization in the prostate tumor environment [94]. Using the exosome pathway, PrCa cells (PC-3) also transfer integrin αVβ6 to β6-negative human microvascular endothelial cells (HMEC1) to promote proliferation, migration and tube formation in endothelial cells [102]. The functional transfer of exosomal integrins is also reported in breast cancer. Recent work has shown that integrin αVβ3 from malignant MDA-MB-231 breast cancer cells is effectively transferred via exosomes to non-malignant MCF10A breast epithelial cells. [118]. Similarly, exosome-associated integrin β4 derived from MDA-MB-231 breast cancer cells is significant in breast cancer progression and the metabolic reprograming of cancer-associated fibroblasts (CAF) in which lactate production and mitophagy are induced by integrin β4 [119]. The role of exosomal integrin αMβ2 has also been noted in the metastasis of hepatocellular carcinoma (HCC), in which the migratory capacity of HCC cells is boosted by exosomal integrin released from M2 macrophages [120]. The involvement of exosomal integrin β1 in the progression of non-alcoholic steatohepatitis is noteworthy. Lipotoxin-treated hepatocytes release many integrin β1-enriched exosomes, which promote the β1-dependent recruitment of proinflammatory monocytes in the liver [121].

ILK is present in exosomes secreted from bone marrow-derived endothelial progenitor cells (EPC). When administered to mouse cardiac endothelial cells (MCEC), ILK-enriched EPC exosomes activate the NF-κB pathway and NF-κB-dependent inflammatory gene expression in recipient cells. Deficiency of anti-inflammatory cytokine IL-10 impairs the myocardial repair process by upregulating the enrichment of ILK in EPC exosomes [122]. Similarly, exosomal transfer of ILK between breast cancer cells and normal mammary epithelial cells is significant in mammary tumorigenesis [123]. Exosomes also transfer a downstream effector of integrins, namely, FAK. Knockdown of integrin β1 in PC3 cells downregulates the level of FAK in their exosomes, which has been implicated in the anchorage-independent growth of PC3 PrCa cells [124]. In the same way, receptor tyrosine kinases (EGFR, HER2), which are carried by breast cancer-derived exosomes, promote the survival of monocytes in inflammatory environments by activating the mitogen-activated protein kinase (MAPK) pathway in monocytes [125].

## 3. Exosome-Mediated Remodeling of Endothelial Gene Expression and Metabolism

### 3.1. Role of Exosomes in Endothelial Metabolism

Endothelial cells that line the innermost part of all blood vessels and lymphatic vessels perform numerous functions, such as the maintenance of vascular integrity, transcellular transport of nutrients and oxygen, leukocyte adhesion, platelet aggregation, interstitial fluid formation and the secretion of vasoactive substances. Bone marrow endothelial cells are involved in the development of hematopoietic stem cells and hematopoiesis [126]. Multiple metabolic pathways, such as glycolysis, fatty acid oxidation and amino acid metabolism, are used by endothelial cells to carry out their functions efficiently [127]. Glycolysis, in which glucose is converted to lactate, rather than oxidative phosphorylation is responsible for the production of 85% of ATP in endothelial cells. 6-phosphofructo-2-kinase/fructose 2,6-bisphosphatase-3 (PFKFB3) and hexokinase 2 (HK2) are the rate-limiting enzymes driving glycolysis in endothelial cells. Fatty acid oxidation, which is mainly controlled by carnitine palmitoyltransferase 1 (CPT1), is necessary for biomass (proteins, lipids, nucleotides) synthesis and redox homeostasis. Metabolism of amino acid glutamine is important for endothelial cells, which are bathed in a high-oxygen environment, in order to withstand oxidative stress [128]. Cysteine metabolism supports endothelial functions, such as cell adhesion, mechano-transduction and flow-induced vasodilatation, by S-sulfhydrating target proteins (e.g., integrins) [129]. Thus, it is not surprising that endothelial cell metabolism is significant for normal physiological processes. Endothelial dysfunction is seen during disease states, such as diabetes mellitus, atherosclerosis, pulmonary arterial hypertension and cancer, as metabolism is perturbed or becomes excessive in these disorders [130].

**Figure 4:**
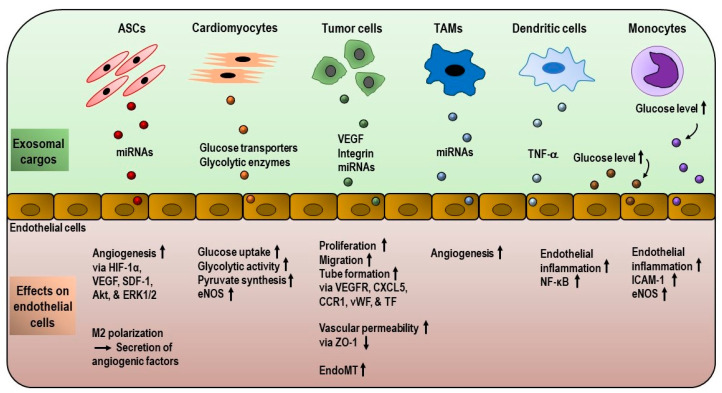
Exosome-mediated remodeling of endothelial gene expression and metabolism. Exosomes derived from various kinds of cells, including tumor and immune cells, have the ability to alter the behavior, metabolism and gene expression in endothelial cells. (↑: increased; ↓: decreased)

Exosomes have an impact on endothelial metabolism and bioenergetics (Figure 4). Exosomes isolated from human umbilical vein endothelial cells (HUVECs) exposed to high glucose levels influence the protein expression and functionality of endothelial cells. They increase the expression of endothelial nitric oxide synthase (eNOS) and ICAM-1 in HUVECs, as well as endothelial wound healing [131]. Metabolism in endothelial cells is influenced by signals from other cell types in their proximity. In this context, rat cardiomyocytes, under glucose deprivation, secrete more exosomes that are loaded with functional glucose transporters (GLUT1, GLUT4) and glycolytic enzymes (lactate dehydrogenase, glyceraldehyde 3-phosphate dehydrogenase). Upon transfer to cardiac microvascular endothelial cells, the internalized exosomes augment glucose uptake, glycolytic activity and pyruvate synthesis in endothelial cells [132]. In addition, cardiomyocyte-derived exosomes activate eNOS, which is responsible for nitric oxide production in endothelial cells, conferring protection against ischemia/reperfusion injuries [133]. This metabolic cross-talk also occurs between endothelial cells and immune cells. Mixed exosomes produced by monocytes and endothelial cells under high glucose conditions favor endothelial inflammation by increasing the endothelial expression of ICAM-1 [134]. Likewise, exosomes derived from mature dendritic cells are involved in the progression of endothelial inflammation via vesicular tumor necrosis factor-α (TNF-α)-mediated NFκB pathways [135]. Oxidized low-density lipoprotein-stimulated macrophages suppress tube formation in endothelial cells via exosomes [136]. Together, these studies provide strong evidence that exosomes can modulate endothelial metabolism and functions.

### 3.2. Role of Exosomes in Angiogenesis and Tumor Microenvironment

Angiogenesis is a complex biological process by which new blood vessels are formed. This process requires coordinated activities, such as the proliferation and migration of endothelial cells. Endothelial cells are quiescent in normal states, but proliferate in a rapid manner under ischemia and hypoxic conditions or in response to injury [128]. During the growth of new vessels, endothelial cells dynamically adapt their metabolism to the increased demands of energy substrates and biomass synthesis. Glycolysis is likely to be the main metabolic pathway used by migrating tip cell phenotypes to sustain energy during vascular sprouting. Fatty acid oxidation provides proliferating stalk cells with the substrates necessary for biomass synthesis. After the formation of new vessel sprouts, endothelial cells differentiate into quiescent phalanx cells in which glycolytic rates and mitochondrial respiration are lower compared to tip cells and stalk cells [137]. Vascular endothelial growth factor (VEGF), fibroblast growth factor (FGF) and other angiogenic signals are key factors in regulating the angiogenic process [128]. In recent years, exosomes have been shown to play a role in the angiogenic process. For instance, exosomes from adipose-derived stem cells (ASCs) promote angiogenesis in vitro, as well as in a mouse model of hindlimb ischemia. They also induce the polarization of M1 macrophages to M2 macrophages, which secrete angiogenic cytokines and growth factors. This study also highlighted the possible synergistic impact of ASC exosomes and M2 macrophages on angiogenesis [138]. Another study also showed that ASC exosomes promote vascularization. They explained the underlying mechanism in which those exosomes that overexpress miR-21 upregulate the expression of hypoxia-inducible factor-1α (HIF-1α), VEGF, stromal cell-derived factor-1 (SDF-1), Akt and the extracellular signal-regulated kinases ERK1/2 in HUVECs [139]. 

Tumor endothelial cells and cancer cells are important components of the tumor microenvironment. Their metabolic cross-talk, dynamic interaction and competition for nutrients influence the progression of cancer [140]. Cancer cells use massive amounts of glucose for their proliferation and survival. At the same time, cancer cells can suppress glucose utilization in neighboring cells by transferring exosomes loaded with miR-122, which mediates the inhibition of glycolytic enzyme pyruvate kinase [141]. Furthermore, cancer cells can absorb metabolic cargos that are carried by CAF-derived exosomes [142]. In hypoxic environments, cancer cells gain the ability to promote cancer progression by increasing the release of exosomes to neighboring cells. Hypoxic breast cancer cells promote tumor spreading by releasing exosomes, which induce mitochondrial reprogramming, ILK-Akt activation and malignant morphogenesis in normal mammary epithelial cells [123]. Meanwhile, hypoxic cancer cells increase the rate of glycolysis, leading to the release of lactate, which can be taken up by nearby cells, including tumor endothelial cells. Unlike normal endothelial cells, tumor endothelial cells exhibit a hyper-proliferative feature, with an increase in glycolysis, fatty acid synthesis, glutamine metabolism and mitochondrial respiration [130]. Therefore, the metabolite lactate secreted by cancer cells is likely to be important for endothelial cells in glucose-deprived tumor environments, leading to abnormal proliferation. Lactate has been shown to play a significant role in promoting angiogenesis by increasing VEGF, HIF-1α and IL-8 signaling in endothelial cells [143]. Previous studies have suggested that the metabolism of hyper-glycolytic tumor endothelial cells can be inhibited, and this blockage has shown advantageous effects in pre-clinical tumor models. For instance, inhibiting PFKFB3 in hyper-glycolytic tumor endothelial cells has beneficial effects, such as tumor vessel normalization, reduced metastasis and improved chemotherapy [144]. Thus, targeting endothelial metabolism can be an alternative option or an additive treatment to suppress tumor angiogenesis, as there are limitations in the efficacy and usefulness of anti-angiogenic drugs (e.g., VEGF antagonists) in the treatment of cancer [130].

In addition to metabolic cross-talk between cancer cells and endothelial cells, they communicate with each other via exosomes. Therefore, it is tempting to focus on how cancer cell-derived exosomes influence endothelial metabolism and angiogenesis, since they can be considered as crucial mediators in tumor microenvironments. Exosomes derived from acute myeloid leukemia (AML) cells influence the activities of HUVECs, such as proliferation, migration and tube formation by enhancing glycolysis, as well as by inducing VEGF receptor expression in endothelial cells [145]. Exosomes from rat pancreatic adenocarcinoma cells can promote the proliferation and migration of rat aortic endothelial cells by regulating the endothelial expression of chemokine CXCL5, chemokine receptor CCR1, VEGF receptor 1, von Willebrand factor and tissue factor. It is worth noting that endothelial–exosome interactions and exosome-mediated angiogenic effects depend on the exosomal expression of the Tspan8-integrin α4β1 complex [110]. The impact of exosome-associated integrins on the angiogenic potential of endothelial cells is also demonstrated in prostate cancer progression. As described above, PrCa exosomes promote angiogenesis by transferring exosomal integrin αVβ6 to endothelial cells, which do not normally express epithelial-specific integrin αVβ6. The uptake of exosomal αVβ6 is related to the upregulation of angiogenesis-promoting survivin levels and the downregulation of angiogenesis-inhibiting pSTAT1 in endothelial cells [102]. Moreover, tumor exosomes can carry and transport VEGF to endothelial cells to promote angiogenesis. Angiogenic effects are mediated via VEGF receptor 2 signaling, and VEGF-bound tumor exosomes confer resistance to treatment with VEGF antibody bevacizumab [146]. It is well known that tumor exosomes favor metastatic spread by preparing pre-metastatic niches. The study by Hoshino et al. demonstrated that pre-metastatic niche formation by different cancer cells follows distinct expression patterns of exosomal integrins [101]. Intriguingly, colorectal cancer-derived exosomes have been implicated in pre-metastatic niche formation by inducing vascular permeability and angiogenesis. These effects are mediated by cancer-promoting miR-25-3p, which is loaded in exosomes [147]. In the same way, exosomes from metastatic breast cancer and hypoxic lung cancer destroy the vascular barrier and promote metastasis by downregulating tight junction proteins, such as zonula occludens-1 (ZO-1) [148,149]. Endothelial-to-mesenchymal transition (EndoMT), in which endothelial cells are differentiated into CAFs, is an important mechanism underlying tumor growth and metastasis. Exosomes from different types of cancer cells can efficiently induce EndoMT, in which endothelial cells undergo significant changes in phenotype, genotype and behavior. CAFs, in turn, gain the ability to remodel the ECM, to disrupt the vascular barrier and to stimulate the migration and invasion of cancer cells [150]. Conversely, mesenchymal stem cell-derived exosomes have the ability to reverse the EndoMT process via the recovery of CAFs back to endothelial cells [151]. Tumor-associated macrophages (TAMs), which are major immune cells in tumor microenvironments, are involved in cancer progression (e.g., HCC) by releasing their exosomes into tumor cells [120]. The specific participation of TAM-exosomes in tumor angiogenesis was shown in one study in which M2-polarized TAMs induced angiogenesis in pancreatic ducal adenocarcinoma by transporting miR-155-5p- and miR-221-5p-loaded exosomes to endothelial cells [152]. Collectively, these data strongly suggest that tumor exosomes have the ability to reprogram endothelial cells within the tumor microenvironment, thereby promoting tumor growth and metastasis.

Another alternative strategy of tumor exosomes to promote tumor progression is mediated through their exosomal expression of programmed death ligands (PDL-1 or PDL-2). Programmed cell death protein 1 (PD-1), which is predominantly expressed in T-cells and tumor-infiltrating lymphocytes, interacts with PDL-1 or PDL-2 to suppress T-cell activation and to induce tumor immune escape [153]. The genetic blockage of exosomal PDL-1 is suggested to confer anti-tumor immunity and to extend the lifespan in a syngeneic mouse model of prostate cancer [154]. In melanoma, tumor cells escape from immunosurveillance by delivering PDL-1 into their exosomes and thereby inhibiting the functions, such as proliferation, cytokine secretion and cytotoxicity of CD8^+^ T-cells. In the clinical setting, the plasma level of exosomal PDL-1 in patients of metastatic melanoma is found to be significantly higher than that of control subjects. Additionally, the level of PDL-1 in circulating exosomes is suggested to be helpful in stratifying clinical responders from non-responders to anti-PDL-1 therapy [155]. Thus, it is prominent that exosomal PDL-1 secreted by tumor cells plays a major role in promoting tumor growth through an immune-dependent mechanism.

## 4. Summary

The diverse functions of cell-adhesion receptor molecule “integrins” are broadened by incorporating their expression into intercellular-communicating nanovesicles called exosomes. The integrins expressed on the surface of exosomes not only facilitate exosomal entry into recipient cells but also help enforce the ability of exosomes to alter the behavior and biologic functions of recipient cells by transferring genetic materials (e.g., miRNAs) or non-genetic materials (e.g., integrins, metabolites and signaling molecules). In addition, exosomal integrins (specifically from cancer and immune cells) are prominently involved in multiple steps of cancer progression. Despite their functional significance, exactly how integrins are regulated in the exosome compartment remains only partially understood. In cellular compartments, it is understood that the active conformational state of integrins is regulated by the cytoskeletal proteins talin and kindlin, although numerous molecules and other factors (e.g., mechanical force) are also involved. There is some speculation that an integrin-regulation mechanism in exosomes might be more or less similar to that at work in cells. Further studies are needed to prove this postulation. Understanding the details of integrin regulation in immune or cancer exosomes, as well as their biologic functions, will provide novel insights for therapeutic strategies targeting immune disorders and malignancies. 

## References

[1] Shimaoka M., Takagi J., Springer T.A. (2002). Conformational Regulation of Integrin Structure and Function. Annu. Rev. Biophys. Biomol. Struct..

[2] Anthis N.J., Campbell I.D. (2011). The tail of integrin activation. Trends Biochem. Sci..

[3] Kim C., Ye F., Ginsberg M.H. (2011). Regulation of Integrin Activation. Annu. Rev. Cell Dev. Biol..

[4] Park E.J., Yuki Y., Kiyono H., Shimaoka M. (2015). Structural basis of blocking integrin activation and deactivation for anti-inflammation. J. Biomed. Sci..

[5] Lafoya B., Munroe J.A., Miyamoto A., Detweiler M.A., Crow J.J., Gazdik T., Albig A.R. (2018). Beyond the matrix: The many non-ECM ligands for integrins. Int. J. Mol. Sci..

[6] Ginsberg M.H. (2014). Integrin activation. BMB Rep..

[7] Shattil S.J., Kim C., Ginsberg M.H. (2010). The final steps of integrin activation: The end game. Nat. Rev. Mol. Cell Biol..

[8] Tadokoro S., Shattil S.J., Eto K., Tai V., Liddington R.C., De Pereda J.M., Ginsberg M.H., Calderwood D.A. (2003). Talin binding to integrin β tails: A final common step in integrin activation. Science.

[9] Gough R.E., Goult B.T. (2018). The tale of two talins—Two isoforms to fine-tune integrin signalling. FEBS Lett..

[10] Calderwood D.A., Zent R., Grant R., Rees D.J.G., Hynes R.O., Ginsberg M.H. (1999). The talin head domain binds to integrin β subunit cytoplasmic tails and regulates integrin activation. J. Biol. Chem..

[11] Calderwood D.A., Yan B., de Pereda J.M., Alvarez B.G., Fujioka Y., Liddington R.C., Ginsberg M.H. (2002). The Phosphotyrosine Binding-like Domain of Talin Activates Integrins. J. Biol. Chem..

[12] Vinogradova O., Velyvis A., Velyviene A., Hu B., Haas T.A., Plow E.F., Qin J. (2002). A structural mechanism of integrin $α$IIb$β$3 “inside-out” activation as regulated by its cytoplasmic face. Cell.

[13] Calderwood D.A. (2015). The Rap1-RIAM pathway prefers β2 integrins. Blood.

[14] Goksoy E., Ma Y., Wang X., Kong X., Perera D., Plow E.F., Qin J. (2008). Structural Basis for the Autoinhibition of Talin in Regulating Integrin Activation. Mol. Cell.

[15] Wang J. (2012). Pull and push: Talin activation for integrin signaling. Cell Res..

[16] Schiemer J., Bohm A., Lin L., Merrill-Skoloff G., Flaumenhaft R., Huang J.S., Le Breton G.C., Chishti A.H. (2016). Gα13 switch region 2 relieves talin autoinhibition to activate αiIbβ3 integrin. J. Biol. Chem..

[17] Rognoni E., Ruppert R., Fässler R. (2016). The kindlin family: Functions, signaling properties and implications for human disease. J. Cell Sci..

[18] Plow E.F., Qin J., Byzova T. (2009). Kindling the flame of integrin activation and function with kindlins. Curr. Opin. Hematol..

[19] Moretti F.A., Moser M., Lyck R., Abadier M., Ruppert R., Engelhardt B., Fassler R. (2013). Kindlin-3 regulates integrin activation and adhesion reinforcement of effector T cells. Proc. Natl. Acad. Sci. USA.

[20] Lefort C.T., Rossaint J., Moser M., Petrich B.G., Zarbock A., Monkley S.J., Critchley D.R., Ginsberg M.H., Fässler R., Ley K. (2012). Distinct roles for talin-1 and kindlin-3 in LFA-1 extension and affinity regulation. Blood.

[21] Wen L., Marki A., Roy P., McArdle S., Sun H., Fan Z., Gingras A.R., Ginsberg M.H., Ley K. (2020). Kindlin-3 recruitment to the plasma membrane precedes high affinity β2 integrin and neutrophil arrest from rolling. Blood.

[22] Margraf A., Germena G., Drexler H.C.A., Rossaint J., Ludwig N., Prystaj B., Mersmann S., Thomas K., Block H., Gottschlich W. (2020). The integrin-linked kinase is required for chemokine-triggered high-affinity conformation of the neutrophil β2-integrin LFA-1. Blood.

[23] Marcovecchio P.M., Zhu Y.P., Hanna R.N., Dinh H.Q., Tacke R., Wu R., McArdle S., Reynolds S., Araujo D.J., Ley K. (2020). Frontline Science: Kindlin-3 is essential for patrolling and phagocytosis functions of nonclassical monocytes during metastatic cancer surveillance. J. Leukoc. Biol..

[24] Gao J., Huang M., Lai J., Mao K., Sun P., Cao Z., Hu Y., Zhang Y., Schulte M.L., Jin C. (2017). Kindlin supports platelet integrin αIIbβ3 activation by interacting with paxillin. J. Cell Sci..

[25] Haydari Z., Shams H., Jahed Z., Mofrad M.R.K. (2020). Kindlin Assists Talin to Promote Integrin Activation. Biophys. J..

[26] Pouwels J., Nevo J., Pellinen T., Ylänne J., Ivaska J. (2012). Negative regulators of integrin activity. J. Cell Sci..

[27] Rantala J.K., Pouwels J., Pellinen T., Veltel S., Laasola P., Mattila E., Potter C.S., Duffy T., Sundberg J.P., Kallioniemi O. (2011). SHARPIN is an endogenous inhibitor of β1-integrin activation. Nat. Cell Biol..

[28] Zeng C., Xiong D., Zhang K., Yao J. (2020). Shank-associated RH domain interactor signaling in tumorigenesis (review). Oncol. Lett..

[29] Gao J., Bao Y., Ge S., Sun P., Sun J., Liu J., Chen F., Han L., Cao Z., Qin J. (2019). Sharpin suppresses β1-integrin activation by complexing with the β1 tail and kindlin-1. Cell Commun. Signal..

[30] Zhang X.A., Hemler M.E. (1999). Interaction of the integrin β1 cytoplasmic domain with ICAP-1 protein. J. Biol. Chem..

[31] Bouvard D., Vignoud L., Dupé-Manet S., Abed N., Fournier H.N., Vincent-Monegat C., Francesco Retta S., Fässler R., Block M.R. (2003). Disruption of focal adhesions by integrin cytoplasmic domain-associated protein-1α. J. Biol. Chem..

[32] Faurobert E., Rome C., Lisowska J., Manet-Dupé S., Boulday G., Malbouyres M., Balland M., Bouin A.P., Kéramidas M., Bouvard D. (2013). CCM1-ICAP-1 complex controls β1 integrin-dependent endothelial contractility and fibronectin remodeling. J. Cell Biol..

[33] Kiema T., Lad Y., Jiang P., Oxley C.L., Baldassarre M., Wegener K.L., Campbell I.D., Ylänne J., Calderwood D.A. (2006). The molecular basis of filamin binding to integrins and competition with talin. Mol. Cell.

[34] Lad Y., Jiang P., Ruskamo S., Harburger D.S., Ylänne J., Campbell I.D., Calderwood D.A. (2008). Structural basis of the migfilin-filamin interaction and competition with integrin β tails. J. Biol. Chem..

[35] Sun Z., Guo S.S., Fässler R. (2016). Integrin-mediated mechanotransduction. J. Cell Biol..

[36] Yakovenko O., Sharma S., Forero M., Tchesnokova V., Aprikian P., Kidd B., Mach A., Vogel V., Sokurenko E., Thomas W.E. (2008). FimH Forms Catch Bonds That Are Enhanced by Mechanical Force Due to Allosteric Regulation. J. Biol. Chem..

[37] Ross T.D., Coon B.G., Yun S., Baeyens N., Tanaka K., Ouyang M., Schwartz M.A. (2013). Integrins in mechanotransduction. Curr. Opin. Cell Biol..

[38] Comrie W.A., Babich A., Burkhardt J.K. (2015). F-actin flow drives affinity maturation and spatial organization of LFA-1 at the immunological synapse. J. Cell Biol..

[39] Nordenfelt P., Elliott H.L., Springer T.A. (2016). Coordinated integrin activation by actin-dependent force during T-cell migration. Nat. Commun..

[40] Li Z., Lee H., Zhu C. (2016). Molecular mechanisms of mechanotransduction in integrin-mediated cell-matrix adhesion. Exp. Cell Res..

[41] Zuidema A., Wang W., Sonnenberg A. (2020). Crosstalk between Cell Adhesion Complexes in Regulation of Mechanotransduction. BioEssays.

[42] Driscoll T.P., Ahn S.J., Huang B., Kumar A., Schwartz M.A. (2020). Actin flow-dependent and -independent force transmission through integrins. Proc. Natl. Acad. Sci. USA.

[43] Lin G.L., Cohen D.M., Desai R.A., Breckenridge M.T., Gao L., Humphries M.J., Chen C.S. (2013). Activation of beta 1 but not beta 3 integrin increases cell traction forces. FEBS Lett..

[44] Milloud R., Destaing O., de Mets R., Bourrin-Reynard I., Oddou C., Delon A., Wang I., Albigès-Rizo C., Balland M. (2017). αvβ3 integrins negatively regulate cellular forces by phosphorylation of its distal NPXY site. Biol. Cell.

[45] Berditchevski F. (2001). Complexes of tetraspanins with integrins: More than meets the eye. J. Cell Sci..

[46] Berditchevski F., Zutter M.M., Hemler M.E. (1996). Characterization of novel complexes on the cell surface between integrins and proteins with 4 transmembrane domains (TM4 proteins). Mol. Biol. Cell.

[47] Kotha J., Longhurst C., Appling W., Jennings L.K. (2008). Tetraspanin CD9 regulates beta 1 integrin activation and enhances cell motility to fibronectin via a PI-3 kinase-dependent pathway. Exp. Cell Res..

[48] Nishiuchi R., Sanzen N., Nada S., Sumida Y., Wada Y., Okada M., Takagi J., Hasegawa H., Sekiguchi K. (2005). Potentiation of the ligand-binding activity of integrin $α$3$β$1 via association with tetraspanin CD151. Proc. Natl. Acad. Sci. USA.

[49] Reyes R., Monjas A., Yánez-Mó M., Cardeñes B., Morlino G., Gilsanz A., Machado-Pineda Y., Lafuente E., Monk P., Sánchez-Madrid F. (2015). Different states of integrin LFA-1 aggregation are controlled through its association with tetraspanin CD9. Biochim. Biophys. Acta Mol. Cell Res..

[50] Shibagaki N., Hanada K.I., Yamashita H., Shimada S., Hamada H. (1999). Overexpression of CD82 on human T cells enhances LFA-1/ICAM-1-mediated cell-cell adhesion: Functional association between CD82 and LFA-1 in T cell activation. Eur. J. Immunol..

[51] Vancompernolle S.E., Levy S., Todd S.C. (2001). Anti-CD81 activates LFA-1 on T cells and promotes T cell-B cell collaboration. Eur. J. Immunol..

[52] Arokiasamy S., Balderstone M.J.M., De Rossi G., Whiteford J.R. (2020). Syndecan-3 in Inflammation and Angiogenesis. Front. Immunol..

[53] Soares M.A., Teixeira F.C.O.B., Fontes M., Arêas A.L., Leal M.G., Pavão M.S.G., Stelling M.P. (2015). Heparan Sulfate Proteoglycans May Promote or Inhibit Cancer Progression by Interacting with Integrins and Affecting Cell Migration. BioMed Res. Int..

[54] Morgan M.R., Humphries M.J., Bass M.D. (2007). Synergistic control of cell adhesion by integrins and syndecans. Nat. Rev. Mol. Cell Biol..

[55] Rovira-Clavé X., Angulo-Ibáñez M., Reina M., Espel E. (2014). The PDZ-binding domain of syndecan-2 inhibits LFA-1 high-affinity conformation. Cell. Signal..

[56] Beauvais D.L.M., Burbach B.J., Rapraeger A.C. (2004). The syndecan-1 ectodomain regulates αvβ3 integrin activily in human mammary carcinoma cells. J. Cell Biol..

[57] Afratis N.A., Nikitovic D., Multhaupt H.A.B., Theocharis A.D., Couchman J.R., Karamanos N.K. (2017). Syndecans—Key regulators of cell signaling and biological functions. FEBS J..

[58] Morgan M.R., Hamidi H., Bass M.D., Warwood S., Ballestrem C., Humphries M.J. (2013). Syndecan-4 Phosphorylation Is a Control Point for Integrin Recycling. Dev. Cell.

[59] De Rossi G., Evans A.R., Kay E., Woodfin A., McKay T.R., Nourshargh S., Whiteford J.R. (2014). Shed syndecan-2 inhibits angiogenesis. J. Cell Sci..

[60] Beauvais D.M., Ell B.J., McWhorter A.R., Rapraeger A.C. (2009). Syndecan-1 regulates α vβ 3 and α vβ 5 integrin activation during angiogenesis and is blocked by synstatin, a novel peptide inhibitor. J. Exp. Med..

[61] Beauvais D.M., Rapraeger A.C. (2010). Syndecan-1 couples the insulin-like growth factor-1 receptor to inside-out integrin activation. J. Cell Sci..

[62] Wang H., Jin H., Beauvais D.M., Rapraeger A.C. (2014). Cytoplasmic Domain Interactions of Syndecan-1 and Syndecan-4 with α6β4 Integrin Mediate Human Epidermal Growth Factor Receptor (HER1 and HER2)-dependent Motility and Survival. J. Biol. Chem..

[63] Fiore V.F., Ju L., Chen Y., Zhu C., Barker T.H. (2014). Dynamic catch of a Thy-1–α5β1+syndecan-4 trimolecular complex. Nat. Commun..

[64] Chronopoulos A., Thorpe S.D., Cortes E., Lachowski D., Rice A.J., Mykuliak V.V., Róg T., Lee D.A., Hytönen V.P., del Río Hernández A.E. (2020). Syndecan-4 tunes cell mechanics by activating the kindlin-integrin-RhoA pathway. Nat. Mater..

[65] Kukkurainen S., Azizi L., Zhang P., Jacquier M.-C., Baikoghli M., von Essen M., Tuukkanen A., Laitaoja M., Liu X., Rahikainen R. (2020). The F1 loop of the talin head domain acts as a gatekeeper in integrin activation and clustering. J. Cell Sci..

[66] Bakker G.J., Eich C., Torreno-Pina J.A., Diez-Ahedo R., Perez-Samper G., Van Zanten T.S., Figdor C.G., Cambi A., Garcia-Parajo M.F. (2012). Lateral mobility of individual integrin nanoclusters orchestrates the onset for leukocyte adhesion. Proc. Natl. Acad. Sci. USA.

[67] Jin T., Li J. (2002). Dynamitin Controls β 2 Integrin Avidity by Modulating Cytoskeletal Constraint on Integrin Molecules. J. Biol. Chem..

[68] Cluzel C., Saltel F., Lussi J., Paulhe F., Imhof B.A., Wehrle-Haller B. (2005). The mechanisms and dynamics of αvβ3 integrin clustering in living cells. J. Cell Biol..

[69] Cambi A., Joosten B., Koopman M., de Lange F., Beeren I., Torensma R., Fransen J.A., Garcia-Parajó M., van Leeuwen F.N., Figdor C.G. (2006). Organization of the Integrin LFA-1 in Nanoclusters Regulates Its Activity. Mol. Biol. Cell.

[70] Yáñez-Mó M., Siljander P.R.-M., Andreu Z., Bedina Zavec A., Borràs F.E., Buzas E.I., Buzas K., Casal E., Cappello F., Carvalho J. (2015). Biological properties of extracellular vesicles and their physiological functions. J. Extracell. Vesicles.

[71] Zaborowski M.P., Balaj L., Breakefield X.O., Lai C.P. (2015). Extracellular Vesicles: Composition, Biological Relevance, and Methods of Study. Bioscience.

[72] Abels E.R., Breakefield X.O. (2016). Introduction to Extracellular Vesicles: Biogenesis, RNA Cargo Selection, Content, Release, and Uptake. Cell. Mol. Neurobiol..

[73] Mashouri L., Yousefi H., Aref A.R., Ahadi A.M., Molaei F., Alahari S.K. (2019). Exosomes: Composition, biogenesis, and mechanisms in cancer metastasis and drug resistance. Mol. Cancer.

[74] Hessvik N.P., Llorente A. (2018). Current knowledge on exosome biogenesis and release. Cell. Mol. Life Sci..

[75] Meldolesi J. (2018). Exosomes and Ectosomes in Intercellular Communication. Curr. Biol..

[76] Lőrincz Á.M., Bartos B., Szombath D., Szeifert V., Timár C.I., Turiák L., Drahos L., Kittel Á., Veres D.S., Kolonics F. (2020). Role of Mac-1 integrin in generation of extracellular vesicles with antibacterial capacity from neutrophilic granulocytes. J. Extracell. Vesicles.

[77] Vicencio J.M., Yellon D.M., Sivaraman V., Das D., Boi-Doku C., Arjun S., Zheng Y., Riquelme J.A., Kearney J., Sharma V. (2015). Plasma Exosomes Protect the Myocardium From Ischemia-Reperfusion Injury. J. Am. Coll. Cardiol..

[78] Frühbeis C., Fröhlich D., Kuo W.P., Amphornrat J., Thilemann S., Saab A.S., Kirchhoff F., Möbius W., Goebbels S., Nave K.-A. (2013). Neurotransmitter-Triggered Transfer of Exosomes Mediates Oligodendrocyte–Neuron Communication. PLoS Biol..

[79] Hur Y.H., Feng S., Wilson K.F., Cerione R.A., Antonyak M.A. (2020). Embryonic Stem Cell-Derived Extracellular Vesicles Maintain ESC Stemness by Activating FAK. Dev. Cell.

[80] Segura E., Nicco C., Lombard B., Véron P., Raposo G., Batteux F., Amigorena S., Théry C. (2005). ICAM-1 on exosomes from mature dendritic cells is critical for efficient naive T-cell priming. Blood.

[81] Segura E., Guérin C., Hogg N., Amigorena S., Théry C. (2007). CD8 + Dendritic Cells Use LFA-1 to Capture MHC-Peptide Complexes from Exosomes In Vivo. J. Immunol..

[82] Wahlgren J., Karlson T.D.L., Glader P., Telemo E., Valadi H. (2012). Activated Human T Cells Secrete Exosomes That Participate in IL-2 Mediated Immune Response Signaling. PLoS ONE.

[83] Torralba D., Baixauli F., Villarroya-Beltri C., Fernández-Delgado I., Latorre-Pellicer A., Acín-Pérez R., Martín-Cófreces N.B., Jaso-Tamame Á.L., Iborra S., Jorge I. (2018). Priming of dendritic cells by DNA-containing extracellular vesicles from activated T cells through antigen-driven contacts. Nat. Commun..

[84] Park E.J., Prajuabjinda O., Soe Z.Y., Darkwah S., Appiah M.G., Kawamoto E., Momose F., Shiku H., Shimaoka M. (2019). Exosomal regulation of lymphocyte homing to the gut. Blood Adv..

[85] Raposo G., Nijman H.W., Stoorvogel W., Liejendekker R., Harding C.V., Melief C.J., Geuze H.J. (1996). B lymphocytes secrete antigen-presenting vesicles. J. Exp. Med..

[86] Jong A.Y., Wu C.-H., Li J., Sun J., Fabbri M., Wayne A.S., Seeger R.C. (2017). Large-scale isolation and cytotoxicity of extracellular vesicles derived from activated human natural killer cells. J. Extracell. Vesicles.

[87] Rajendran L., Honsho M., Zahn T.R., Keller P., Geiger K.D., Verkade P., Simons K. (2006). Alzheimer’s disease beta-amyloid peptides are released in association with exosomes. Proc. Natl. Acad. Sci. USA.

[88] Grey M., Dunning C.J., Gaspar R., Grey C., Brundin P., Sparr E., Linse S. (2015). Acceleration of α-Synuclein Aggregation by Exosomes. J. Biol. Chem..

[89] Singh A., Fedele C., Lu H., Nevalainen M.T., Keen J.H., Languino L.R. (2016). Exosome-mediated Transfer of avb3 Integrin from Tumorigenic to Nontumorigenic Cells Promotes a Migratory Phenotype. Mol. Cancer Res..

[90] Tang M.K.S., Yue P.Y.K., Ip P.P., Huang R.-L., Lai H.-C., Cheung A.N.Y., Tse K.Y., Ngan H.Y.S., Wong A.S.T. (2018). Soluble E-cadherin promotes tumor angiogenesis and localizes to exosome surface. Nat. Commun..

[91] Yu S., Liu C., Su K., Wang J., Liu Y., Zhang L., Li C., Cong Y., Kimberly R., Grizzle W.E. (2007). Tumor Exosomes Inhibit Differentiation of Bone Marrow Dendritic Cells. J. Immunol..

[92] Gaballa R., Ali H.E.A., Mahmoud M.O., Rhim J.S., Ali H.I., Salem H.F., Saleem M., Kandeil M.A., Ambs S., Abd Elmageed Z.Y. (2020). Exosomes-Mediated Transfer of Itga2 Promotes Migration and Invasion of Prostate Cancer Cells by Inducing Epithelial-Mesenchymal Transition. Cancers.

[93] Krishn S.R., Singh A., Bowler N., Duffy A.N., Friedman A., Fedele C., Kurtoglu S., Tripathi S.K., Wang K., Hawkins A. (2019). Prostate cancer sheds the αvβ3 integrin in vivo through exosomes. Matrix Biol..

[94] Lu H., Bowler N., Harshyne L.A., Craig Hooper D., Krishn S.R., Kurtoglu S., Fedele C., Liu Q., Tang H.-Y., Kossenkov A.V. (2018). Exosomal αvβ6 integrin is required for monocyte M2 polarization in prostate cancer. Matrix Biol..

[95] Zhao J., Schlößer H.A., Wang Z., Qin J., Li J., Popp F., Popp M.C., Alakus H., Chon S.-H., Hansen H.P. (2019). Tumor-Derived Extracellular Vesicles Inhibit Natural Killer Cell Function in Pancreatic Cancer. Cancers.

[96] Klibi J., Niki T., Riedel A., Pioche-Durieu C., Souquere S., Rubinstein E., Le Moulec S., Guigay J., Hirashima M., Guemira F. (2009). Blood diffusion and Th1-suppressive effects of galectin-9–containing exosomes released by Epstein-Barr virus–infected nasopharyngeal carcinoma cells. Blood.

[97] Costa-Silva B., Aiello N.M., Ocean A.J., Singh S., Zhang H., Thakur B.K., Becker A., Hoshino A., Mark M.T., Molina H. (2015). Pancreatic cancer exosomes initiate pre-metastatic niche formation in the liver. Nat. Cell Biol..

[98] Melo S.A., Luecke L.B., Kahlert C., Fernandez A.F., Gammon S.T., Kaye J., LeBleu V.S., Mittendorf E.A., Weitz J., Rahbari N. (2015). Glypican-1 identifies cancer exosomes and detects early pancreatic cancer. Nature.

[99] Hornick N.I., Huan J., Doron B., Goloviznina N.A., Lapidus J., Chang B.H., Kurre P. (2015). Serum Exosome MicroRNA as a Minimally-Invasive Early Biomarker of AML. Sci. Rep..

[100] Whiteside T.L. (2016). Tumor-Derived Exosomes and Their Role in Cancer Progression. Adv. Clin. Chem..

[101] Hoshino A., Costa-Silva B., Shen T.-L., Rodrigues G., Hashimoto A., Tesic Mark M., Molina H., Kohsaka S., Di Giannatale A., Ceder S. (2015). Tumour exosome integrins determine organotropic metastasis. Nature.

[102] Krishn S.R., Salem I., Quaglia F., Naranjo N.M., Agarwal E., Liu Q., Sarker S., Kopenhaver J., McCue P.A., Weinreb P.H. (2020). The αvβ6 integrin in cancer cell-derived small extracellular vesicles enhances angiogenesis. J. Extracell. Vesicles.

[103] Soe Z.Y., Prajuabjinda O., Myint P.K., Gaowa A., Kawamoto E., Park E.J., Shimaoka M. (2019). Talin-2 regulates integrin functions in exosomes. Biochem. Biophys. Res. Commun..

[104] Fedele C., Singh A., Zerlanko B.J., Iozzo R.V., Languino L.R. (2015). The αvβ6 Integrin Is Transferred Intercellularly via Exosomes. J. Biol. Chem..

[105] Subramanian B.C., Melis N., Chen D., Wang W., Gallardo D., Weigert R., Parent C.A. (2020). The LTB4–BLT1 axis regulates actomyosin and β2-integrin dynamics during neutrophil extravasation. J. Cell Biol..

[106] Genschmer K.R., Russell D.W., Lal C., Szul T., Bratcher P.E., Noerager B.D., Abdul Roda M., Xu X., Rezonzew G., Viera L. (2019). Activated PMN Exosomes: Pathogenic Entities Causing Matrix Destruction and Disease in the Lung. Cell.

[107] Kawamoto E., Masui-Ito A., Eguchi A., Soe Z.Y., Prajuabjinda O., Darkwah S., Park E.J., Imai H., Shimaoka M. (2019). Integrin and PD-1 Ligand Expression on Circulating Extracellular Vesicles in Systemic Inflammatory Response Syndrome and Sepsis. SHOCK.

[108] Domenis R., Marino M., Cifù A., Scardino G., Curcio F., Fabris M. (2020). Circulating exosomes express α4β7 integrin and compete with CD4+ T cells for the binding to Vedolizumab. PLoS ONE.

[109] Kowal J., Arras G., Colombo M., Jouve M., Morath J.P., Primdal-Bengtson B., Dingli F., Loew D., Tkach M., Théry C. (2016). Proteomic comparison defines novel markers to characterize heterogeneous populations of extracellular vesicle subtypes. Proc. Natl. Acad. Sci. USA.

[110] Nazarenko I., Rana S., Baumann A., McAlear J., Hellwig A., Trendelenburg M., Lochnit G., Preissner K.T., Zoller M. (2010). Cell Surface Tetraspanin Tspan8 Contributes to Molecular Pathways of Exosome-Induced Endothelial Cell Activation. Cancer Res..

[111] Wiklander O.P.B., Nordin J.Z., O’Loughlin A., Gustafsson Y., Corso G., Mäger I., Vader P., Lee Y., Sork H., Seow Y. (2015). Extracellular vesicle in vivo biodistribution is determined by cell source, route of administration and targeting. J. Extracell. Vesicles.

[112] Mulcahy L.A., Pink R.C., Carter D.R.F. (2014). Routes and mechanisms of extracellular vesicle uptake. J. Extracell. Vesicles.

[113] Chen L., Brigstock D.R. (2016). Integrins and heparan sulfate proteoglycans on hepatic stellate cells (HSC) are novel receptors for HSC-derived exosomes. FEBS Lett..

[114] Clayton A., Turkes A., Dewitt S., Steadman R., Mason M.D., Hallett M.B. (2004). Adhesion and signaling by B cell-derived exosomes: The role of integrins. FASEB J..

[115] Fuentes P., Sesé M., Guijarro P.J., Emperador M., Sánchez-Redondo S., Peinado H., Hümmer S., Ramón y Cajal S. (2020). ITGB3-mediated uptake of small extracellular vesicles facilitates intercellular communication in breast cancer cells. Nat. Commun..

[116] Yuan D., Zhao Y., Banks W.A., Bullock K.M., Haney M., Batrakova E., Kabanov A.V. (2017). Macrophage exosomes as natural nanocarriers for protein delivery to inflamed brain. Biomaterials.

[117] Wubbolts R., Leckie R.S., Veenhuizen P.T.M., Schwarzmann G., Möbius W., Hoernschemeyer J., Slot J.-W., Geuze H.J., Stoorvogel W. (2003). Proteomic and Biochemical Analyses of Human B Cell-derived Exosomes. J. Biol. Chem..

[118] Altei W.F., Pachane B.C., dos Santos P.K., Ribeiro L.N.M., Sung B.H., Weaver A.M., Selistre-de-Araújo H.S. (2020). Inhibition of αvβ3 integrin impairs adhesion and uptake of tumor-derived small extracellular vesicles. Cell Commun. Signal..

[119] Sung J.S., Kang C.W., Kang S., Jang Y., Chae Y.C., Kim B.G., Cho N.H. (2020). ITGB4-mediated metabolic reprogramming of cancer-associated fibroblasts. Oncogene.

[120] Wu J., Gao W., Tang Q., Yu Y., You W., Wu Z., Fan Y., Zhang L., Wu C., Han G. (2020). M2 macrophage-derived exosomes facilitate hepatocarcinoma metastasis by transferring αMβ2 integrin to tumor cells. Hepatology.

[121] Guo Q., Furuta K., Lucien F., Gutierrez Sanchez L.H., Hirsova P., Krishnan A., Kabashima A., Pavelko K.D., Madden B., Alhuwaish H. (2019). Integrin β1-enriched extracellular vesicles mediate monocyte adhesion and promote liver inflammation in murine NASH. J. Hepatol..

[122] Yue Y., Wang C., Benedict C., Huang G., Truongcao M., Roy R., Cimini M., Garikipati V.N.S., Cheng Z., Koch W.J. (2020). Interleukin-10 Deficiency Alters Endothelial Progenitor Cell–Derived Exosome Reparative Effect on Myocardial Repair via Integrin-Linked Kinase Enrichment. Circ. Res..

[123] Bertolini I., Ghosh J.C., Kossenkov A.V., Mulugu S., Krishn S.R., Vaira V., Qin J., Plow E.F., Languino L.R., Altieri D.C. (2020). Small Extracellular Vesicle Regulation of Mitochondrial Dynamics Reprograms a Hypoxic Tumor Microenvironment. Dev. Cell.

[124] DeRita R.M., Sayeed A., Garcia V., Krishn S.R., Shields C.D., Sarker S., Friedman A., McCue P., Molugu S.K., Rodeck U. (2019). Tumor-Derived Extracellular Vesicles Require β1 Integrins to Promote Anchorage-Independent Growth. iScience.

[125] Song X., Ding Y., Liu G., Yang X., Zhao R., Zhang Y., Zhao X., Anderson G.J., Nie G. (2016). Cancer Cell-derived Exosomes Induce Mitogen-activated Protein Kinase-dependent Monocyte Survival by Transport of Functional Receptor Tyrosine Kinases. J. Biol. Chem..

[126] Itkin T., Gur-Cohen S., Spencer J.A., Schajnovitz A., Ramasamy S.K., Kusumbe A.P., Ledergor G., Jung Y., Milo I., Poulos M.G. (2016). Distinct bone marrow blood vessels differentially regulate haematopoiesis. Nature.

[127] Li X., Kumar A., Carmeliet P. (2019). Metabolic Pathways Fueling the Endothelial Cell Drive. Annu. Rev. Physiol..

[128] Rohlenova K., Veys K., Miranda-Santos I., De Bock K., Carmeliet P. (2018). Endothelial Cell Metabolism in Health and Disease. Trends Cell Biol..

[129] Bibli S.-I., Hu J., Looso M., Weigert A., Ratiu C., Wittig J., Drekolia M.K., Tombor L., Randriamboavonjy V., Leisegang M.S. (2020). Mapping the Endothelial Cell S -Sulfhydrome Highlights the Crucial Role of Integrin Sulfhydration in Vascular Function. Circulation.

[130] Li X., Sun X., Carmeliet P. (2019). Hallmarks of Endothelial Cell Metabolism in Health and Disease. Cell Metab..

[131] Sáez T., de Vos P., Kuipers J., Sobrevia L., Faas M.M. (2018). Fetoplacental endothelial exosomes modulate high d-glucose-induced endothelial dysfunction. Placenta.

[132] Garcia N.A., Moncayo-Arlandi J., Sepulveda P., Diez-Juan A. (2016). Cardiomyocyte exosomes regulate glycolytic flux in endothelium by direct transfer of GLUT transporters and glycolytic enzymes. Cardiovasc. Res..

[133] Chen G., Xu C., Gillette T.G., Huang T., Huang P., Li Q., Li X., Li Q., Ning Y., Tang R. (2020). Cardiomyocyte-derived small extracellular vesicles can signal eNOS activation in cardiac microvascular endothelial cells to protect against Ischemia/Reperfusion injury. Theranostics.

[134] Sáez T., de Vos P., Kuipers J., Sobrevia L., Faas M.M. (2019). Exosomes derived from monocytes and from endothelial cells mediate monocyte and endothelial cell activation under high d-glucose conditions. Immunobiology.

[135] Gao W., Liu H., Yuan J., Wu C., Huang D., Ma Y., Zhu J., Ma L., Guo J., Shi H. (2016). Exosomes derived from mature dendritic cells increase endothelial inflammation and atherosclerosis via membrane TNF-α mediated NF-κB pathway. J. Cell. Mol. Med..

[136] Huang C., Huang Y., Zhou Y., Nie W., Pu X., Xu X., Zhu J. (2018). Exosomes derived from oxidized LDL-stimulated macrophages attenuate the growth and tube formation of endothelial cells. Mol. Med. Rep..

[137] Dumas S.J., García-Caballero M., Carmeliet P. (2020). Metabolic Signatures of Distinct Endothelial Phenotypes. Trends Endocrinol. Metab..

[138] Zhu D., Johnson T.K., Wang Y., Thomas M., Huynh K., Yang Q., Bond V.C., Chen Y.E., Liu D. (2020). Macrophage M2 polarization induced by exosomes from adipose-derived stem cells contributes to the exosomal proangiogenic effect on mouse ischemic hindlimb. Stem Cell Res. Ther..

[139] An Y., Zhao J., Nie F., Qin Z., Xue H., Wang G., Li D. (2019). Exosomes from Adipose-Derived Stem Cells (ADSCs) Overexpressing miR-21 Promote Vascularization of Endothelial Cells. Sci. Rep..

[140] Yang E., Wang X., Gong Z., Yu M., Wu H., Zhang D. (2020). Exosome-mediated metabolic reprogramming: The emerging role in tumor microenvironment remodeling and its influence on cancer progression. Signal Transduct. Target. Ther..

[141] Fong M.Y., Zhou W., Liu L., Alontaga A.Y., Chandra M., Ashby J., Chow A., O’Connor S.T.F., Li S., Chin A.R. (2015). Breast-cancer-secreted miR-122 reprograms glucose metabolism in premetastatic niche to promote metastasis. Nat. Cell Biol..

[142] Zhao H., Yang L., Baddour J., Achreja A., Bernard V., Moss T., Marini J.C., Tudawe T., Seviour E.G., San Lucas F.A. (2016). Tumor microenvironment derived exosomes pleiotropically modulate cancer cell metabolism. Elife.

[143] Harjes U., Bensaad K., Harris A.L. (2012). Endothelial cell metabolism and implications for cancer therapy. Br. J. Cancer.

[144] Cantelmo A.R., Conradi L.-C., Brajic A., Goveia J., Kalucka J., Pircher A., Chaturvedi P., Hol J., Thienpont B., Teuwen L.-A. (2016). Inhibition of the Glycolytic Activator PFKFB3 in Endothelium Induces Tumor Vessel Normalization, Impairs Metastasis, and Improves Chemotherapy. Cancer Cell.

[145] Wang B., Wang X., Hou D., Huang Q., Zhan W., Chen C., Liu J., You R., Xie J., Chen P. (2019). Exosomes derived from acute myeloid leukemia cells promote chemoresistance by enhancing glycolysis-mediated vascular remodeling. J. Cell. Physiol..

[146] Ko S.Y., Lee W., Kenny H.A., Dang L.H., Ellis L.M., Jonasch E., Lengyel E., Naora H. (2019). Cancer-derived small extracellular vesicles promote angiogenesis by heparin-bound, bevacizumab-insensitive VEGF, independent of vesicle uptake. Commun. Biol..

[147] Zeng Z., Li Y., Pan Y., Lan X., Song F., Sun J., Zhou K., Liu X., Ren X., Wang F. (2018). Cancer-derived exosomal miR-25-3p promotes pre-metastatic niche formation by inducing vascular permeability and angiogenesis. Nat. Commun..

[148] Zhou W., Fong M.Y., Min Y., Somlo G., Liu L., Palomares M.R., Yu Y., Chow A., O’Connor S.T.F., Chin A.R. (2014). Cancer-Secreted miR-105 Destroys Vascular Endothelial Barriers to Promote Metastasis. Cancer Cell.

[149] Hsu Y.-L., Hung J.-Y., Chang W.-A., Lin Y.-S., Pan Y.-C., Tsai P.-H., Wu C.-Y., Kuo P.-L. (2017). Hypoxic lung cancer-secreted exosomal miR-23a increased angiogenesis and vascular permeability by targeting prolyl hydroxylase and tight junction protein ZO-1. Oncogene.

[150] Kim K., Sohn Y.J., Lee R., Yoo H.J., Kang J.Y., Choi N., Na D., Yeon J.H. (2020). Cancer-Associated Fibroblasts Differentiated by Exosomes Isolated from Cancer Cells Promote Cancer Cell Invasion. Int. J. Mol. Sci..

[151] Yeon J.H., Jeong H.E., Seo H., Cho S., Kim K., Na D., Chung S., Park J., Choi N., Kang J.Y. (2018). Cancer-derived exosomes trigger endothelial to mesenchymal transition followed by the induction of cancer-associated fibroblasts. Acta Biomater..

[152] Yang Y., Guo Z., Chen W., Wang X., Cao M., Han X., Zhang K., Teng B., Cao J., Wu W. (2020). M2 Macrophage-Derived Exosomes Promote Angiogenesis and Growth of Pancreatic Ductal Adenocarcinoma by Targeting E2F2. Mol. Ther..

[153] Nakamori Y., Park E.J., Shimaoka M. (2021). Immune Deregulation in Sepsis and Septic Shock: Reversing Immune Paralysis by Targeting PD-1/PD-L1 Pathway. Front. Immunol..

[154] Poggio M., Hu T., Pai C.-C., Chu B., Belair C.D., Chang A., Montabana E., Lang U.E., Fu Q., Fong L. (2019). Suppression of Exosomal PD-L1 Induces Systemic Anti-tumor Immunity and Memory. Cell.

[155] Chen G., Huang A.C., Zhang W., Zhang G., Wu M., Xu W., Yu Z., Yang J., Wang B., Sun H. (2018). Exosomal PD-L1 contributes to immunosuppression and is associated with anti-PD-1 response. Nature.

